# miR-141 and miR-200a, Revelation of New Possible Players in Modulation of Th17/Treg Differentiation and Pathogenesis of Multiple Sclerosis

**DOI:** 10.1371/journal.pone.0124555

**Published:** 2015-05-04

**Authors:** Reza Naghavian, Kamran Ghaedi, Abbas Kiani-Esfahani, Mazdak Ganjalikhani-Hakemi, Masoud Etemadifar, Mohammad Hossein Nasr-Esfahani

**Affiliations:** 1 Division of Cellular and Molecular Biology, Department of Biology, Faculty of Sciences, University of Isfahan, Isfahan, Iran; 2 Department of Cellular Biotechnology at Cell Science research Center, Royan Institute for Biotechnology, ACECR, Isfahan, Iran; 3 Department of Neurology, School of Medicine, Isfahan University of Medical Sciences, Isfahan, Iran; 4 Multiple Sclerosis and Neuroimmunology Research Center, Isfahan, Iran; 5 Cellular and Molecular Immunology Research Center, Faculty of Medicine, Isfahan University of Medical Sciences, Isfahan, Iran; 6 Immunology Department, School of Medicine, Isfahan University of Medical Sciences, Isfahan, Iran; Institut Pasteur, FRANCE

## Abstract

**Background:**

One of the main issues in pathogenesis of MS is Th17/Treg imbalance. There are growing interests in nominating miRNAs involved in Th17 cell differentiation, suggesting them as new therapeutic agents that may reduce progression of different autoimmune diseases specially MS.

**Objectives:**

We assessed transcript levels of miR-141 and miR-200a in MS patients, during relapsing and remitting phases. We also investigated possible role of miR-141, miR-200a in inducing differentiation to Th17 cells.

**Materials and Methods:**

Forty RR-MS patient samples including relapsing (n=20) and remitting (n=20) phases were chosen. Expression level of miR-141 and miR-200a were measured by RT-q PCR and compared to healthy control group (n=10). *In-silico* analyses on miR-141 and miR-200a targetome showed involvement of both miRNAs in T helper cell differentiation pathways including TGF-β, mTOR and JAK/STAT.

**Results:**

We observed that percentage of RORγt^+^ CD4^+^ T cells increase in relapsing phase while FOXP3^+^ CD4^+^ increase in remitting phase of MS patients. Furthermore, both miR-141 and miR-200a show up-regulation in relapsing phase of MS patients compared to remitting and control groups. Interestingly, expression level of target genes of miR-141 and miR-200a, which were assessed through *in-silico* methods, show down-regulation in relapsing phase of MS patients.

**Conclusions:**

According to our results, miR-141 and miR-200a may be key miRNAs in progression of symptoms of MS through inducing differentiation of Th17 cells and inhibiting differentiation to Treg cells. Our data suggest that these miRNAs may probably inhibit negative regulators of Th17 cell differentiation, thus promoting its differentiation.

## Introduction

Multiple Sclerosis (MS) is a neurodegenerative chronic autoimmune disease of the CNS in which myelin and axons are destroyed to different degrees [1]. Although development of MS is highly irregular, it is mostly considered by occurrence of reversible neurological malfunctions which deteriorates over time.

Epidemiology of MS in developing countries as Iran and large cities as Isfahan displays that there is a wild growth in frequency of affected patients with an overall prevalence of 85.8 per 100000 [2]. MS is known to be a multifactorial disease with still no definite cause but it appears that combination of environmental factors, epigenetic and genetics lead to continuing immune attacks on the CNS [3].

Primarily it was supposed that a subset of CD4^+^ T cells with a Th1 phenotype producing IFN-γ is critical in autoimmunity of MS, however it is now clear that IL-17 producing CD4^+^ T cells, known as Th17, are the main responsible cells for inflammation and pathogenesis of MS [4]. Th17 cells mainly do their effects through secreting IL-17, IL-21, IL-22 and GM-CSF, which are essential for autoimmune neuro-inflammation [5,6]. Generally, activation of different STAT transcription factors along with master regulator of each lineage leads to differentiation of various CD4^+^ T cell subtypes. Following the activation of specific transcription factors of STAT3/RORγt, naïve CD4^+^ T cells differentiate to Th17 [7,8]. Various studies indicate up-regulation of Th17 cells in different autoimmune diseases such as MS and experimental autoimmune encephalomyelitis (EAE). Furthermore, Tzartos *et al*. showed that expression level of IL-17 increases in active lesions of MS patients [9,10]. In addition, as well as other autoimmune diseases, MS is closely related to deficiencies in immune regulation through malfunction or decreased number of regulatory T cells (Treg) [4] and its differentiation occurs through activation of *STAT5*/ *FOXP3* [8].

Various studies showed that microRNAs (miRNAs) play significant roles in different processes including hematopoiesis and function of diverse sets of immune cells such as T cells through suppressing different mRNAs in post-transcriptional level [11,12].

miR-200 family includes two cluster of miRNAs which one is on chromosome 1p36.3 (miR-200a/200b/429) and its members have the same seed length (AAUACU (while the other one is on chromosome 12p13 (miR-141/200c) and its members have the same seed length (AACACU) which is highly similar to cluster one [13].

Different studies including our previous study [14], have explored deregulation of different miRNAs in peripheral blood mononuclear cells (PBMC), B cells, CD4^+^ T cells and tissues of MS patients, so far. Furthermore, several studies have also investigated the role of different miRNAs on differentiation of Th17 cells. Studies on miR-141 and miR-200a have shown that these miRNAs are involved in different autoimmune diseases, although their role in different cancers is well known as well. Studies on systemic lupus erythematosus (SLE), inflammatory bowel disease (IBD), psoriasis and other immune-related diseases display deregulation of miR-141 and miR-200a [15–17]. Despite aforementioned studies, miR-141 and miR-200 were never studied or focused on in MS patients and T helper cell differentiation before. The initial notion is that in case of miR-141 and miR-200a’s role in differentiation of Th17 cells, they should display up-regulation consistent with increase in the number of Th17 cells in relapsing phase of MS. Inversely they should indicate down-regulation with reduction of Th17 cells in remitting phase.

The aim of the current study is to evaluate miR-141 and miR-200a expression in CD4^+^ T cells of relapsing versus remitting phase of MS patients; through which we tried to find their possible role in differentiation or suppression of Th17 and/or Treg cells by assessing the correlation between them and the percentage of these cells in different phases of MS. Furthermore, possible targets of miR-141 and miR-200a that could be involved in differentiation of T helper cells were established *in-silico*, and their accuracy was investigated. Therefore, concluding intention of this study is to suggest miR-141 and miR-200a as novel miRNAs involved in differentiation of Th17 cells, thereby pathogenesis of MS.

## Materials and Methods

### Patients and control samples

Total blood samples (10 ml with EDTA) were drawn from 40 patients with relapsing-remitting MS (RRMS) attending Al-Zahra Hospital (Isfahan, Iran), including 20 newly diagnosed (as relapsing phase) and 20 patients in remitting phase. Ten samples were also taken from healthy volunteers. Patients were diagnosed based on McDonald criteria. All patients in relapsing phase had no drug treatment while all patients in remitting phase were chosen among those persons who were only treated with Synovex (IFN-β) to avoid drug complication effects. Sampling were done a week after previous injection and just before the next shot to minimize the drug effects. Written informed consent was obtained from all studied subjects. All study protocols and written consent forms taken from the individuals in this study were approved by institutional review board of Royan Institute (Project Id. No. 91000573). Written consent forms are recorded and available in Royan institute archives. All clinical information of patients including age, sex, disease duration and MRI results are signified in S1 Table.

### Cell isolation

PBMC were isolated using density gradient lymphoprep (STEMCELL Technologies, USA) according to manufacturer’s instruction. Cell subpopulations, CD4^+^ T cells, were isolated from PBMC with CD4^+^ T cell isolation kit II human of Miltenyi Biotec (Miltenyi Biotec, Bergisch Gladbach, Germany), with purity of more than 95 percent [18] and according to manufacturer’s instruction.

### Flow Cytometry

To quantify RORγt and FOXP3 levels as Th17 and Treg cells markers respectively, flow cytometry was performed. At the first step, the isolated cells were fixed and stained with the following antibodies: Anti-Human Foxp3 PE, Anti-Human/Mouse ROR γ(t) PE, Anti-Human CD4 Alexa Fluor 488 and analyzed on Becton Dickinson FACSCalibur flow cytometer (USA). Data were analyzed by comparison of the Mouse IgG1 K Isotype Control PE, Rat IgG2a K Isotype Control PE, Mouse IgG2b K Isotype Control Alexa Fluor 488(all antibodies were purchased from eBioscience, USA). A forward and side scatter gate was used to select lymphocyte population and fluorescence compensation was set according to labeled lymphocytes with only green and only red fluorescence separately versus isotype control. Then, the samples were run in acquisition phase. Green fluorescence from FITC-conjugated antibodies and red fluorescence from PE-conjugated antibodies were collected in fluorescence detector 1 and 2 respectively. Percentages of Th-17 and Treg cells were determined after gating without debris and aggregate. CellQuest Pro software was used to analyze data.

### RNA extraction

Total RNA (including miRNA) were isolated by TRizol reagent (Invitrogen,USA) according to manufacturer’s instruction.

### cDNA synthesis and real-time PCR

cDNA synthesis for miR-141 and miR-200a was achieved by using a “universal cDNA synthesis kit” (Exiqon, Denmark) in a poly A tailing method, as stated by manufacturer. Real-time quantitative PCR (RT-qPCR) reactions were performed using standard protocol with an ABI PRISM 7500 instrument (Applied Biosystems, USA); cDNA products were added to a master mix comprising 10 pmol/μl of each miR-141 or miR-200a DNA primers (Exeqon, Denmark) and 2 U of SYBR premix ExTaq II (TaKaRa, Japan), similar to our previous study [14]. *RNU48* small nucleolar RNA was quantified as the reference to normalize differences in total RNA levels. cDNA synthesis for *IL-17A*, *IL-23R*, *RORC*, *TGF-β*, *FOXP3*, *SMAD2*, *GATA3* and *FOXO3* was carried out on a total RNA which has reverse-transcribed by RevertAid First Strand cDNA synthesis Kit (Thermo Scientific, USA) using random hexamer primers. RT-qPCR was carried out using specific primer pairs in ABI PRISM 7500 instrument (Applied Biosystems, USA). The expression level of these genes was normalized by *18srRNA* as reference gene. All reactions were carried out in triplicate. Real-time data were evaluated and reported based on ΔΔCT method.

### Poly Acrylamide gel & T/A cloning

To evaluate the specificity of miR-141 and miR-200a primers, real time PCR products were run on 12% poly acrylamide gel to see solo amplified band with length of 80bp. This band was extracted from the gel and T/A cloned into pTZ57R/T vector (Thermo Scientific) and were send to sequence.

### Statistical analysis

All statistical tests were executed by SPSS (version 20). Comparison between groups was analyzed by statistical ANOVA test and followed by analyzing with nonparametric Mann—Whitney post-hoc *t*-test. Data are presented as mean ± SEM and considered significant at p < 0.05.

### Molecular signaling pathway enrichment analysis

In order to accomplish molecular enrichment analysis on miR-141 and miR-200a targetome and to find most related signaling pathways which might be involved, we used online *in-silico* databases of miRWalk [18] and miRTarBase [19] to attain predicted and validated targets of miR-141 and miR-200a respectively. We also used DIANA miRPath v.2.0 [20] to visualize pathways of validated targets of miR-141 and miR-200a as a heatmap. Next, using UniGene database (http://www.ncbi.nlm.nih.gov/unigene/) and subsequent EST profile, we investigated their expression in lymph node and thymus since it will be indispensable if they are involved in differentiation of T helper cells. Finally, miR-141 and miR-200a targetome expressed in lymph nodes and thymus were assigned to database for annotation, visualization and integrated discovery (DAVID) bioinformatics database [21] to categorize the most applicable pathways and molecular networks with miR-141 and miR-200a targetome.

## Results

### Number of Th17 cells increased significantly in relapsing phase of MS, while number of Treg cells increased in remission phase of MS

**Fig 1:**
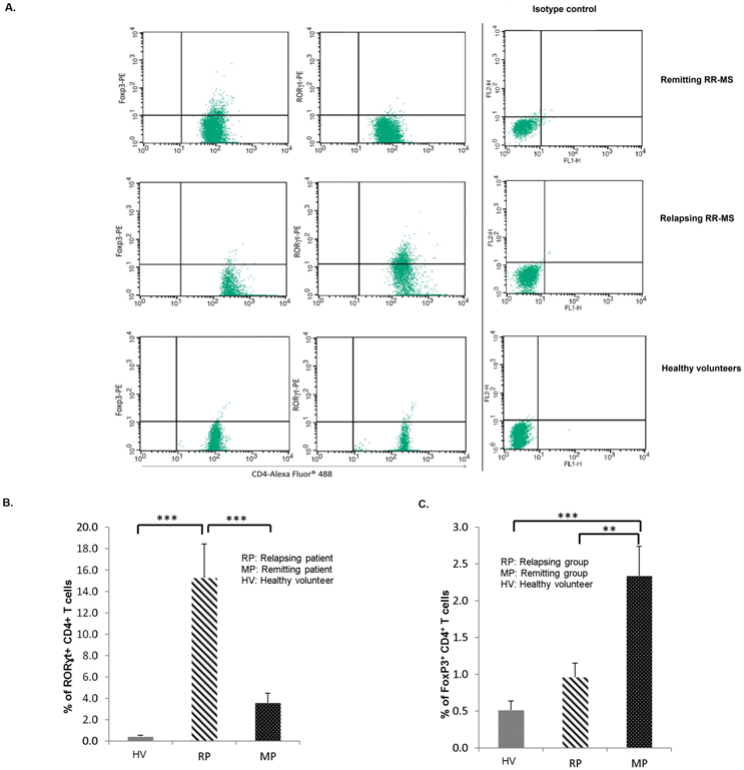
Flow cytometry of FoxP3^+^ CD4^+^ T cells and RORɣt+ CD4+ T cells. CD4^**+**^ T cells were isolated by CD4+ Tcell isolation kit II human of Miltenyi Biotec and stained with respective antibodies and evaluated in relapsing phase (n = 20) and remitting phase (n = 20) of MS patients and healthy controls (n = 10). A forward and side scatter gate was used to select lymphocyte population and fluorescence compensation was set according to labeled lymphocytes with only green and only red fluorescent separately versus isotype control (*A*). Percentage of RORγt+ CD4+ T cells measured by Flow cytometry, shows meaningful increase in relapsing group (*B*) while percentage of FoxP3^**+**^ CD4^**+**^ T cells elevates in remitting group (*C*) (*p < 0.05, **p < 0.01 and ***p < 0.005, non-parametric Mann-Whitney *t*-test) (RP: Relapsing patient, MP: Remitting patient, HV: Healthy volunteer).

First, to evaluate the number of Th17 and Treg cells in different phases of MS, part of CD4^+^ T cells yielded from MACS were stained with FoxP3, RORγt and CD4 antibodies as explained in materials and methods. Results showed that percentage of RORγt^+^ CD4^+^ T cells significantly increased in relapsing versus remitting and control group (p value = 0.0002, p value = 0.0003 respectively), while percentage of FoxP3^+^ CD4^+^ T cells significantly increased in remitting compared to relapsing and healthy controls (p value = 0.003, p value = 0.001 respectively) (Fig 1A–1C).

### Up-regulation of miR-141 in relapsing phase of MS

**Fig 2:**
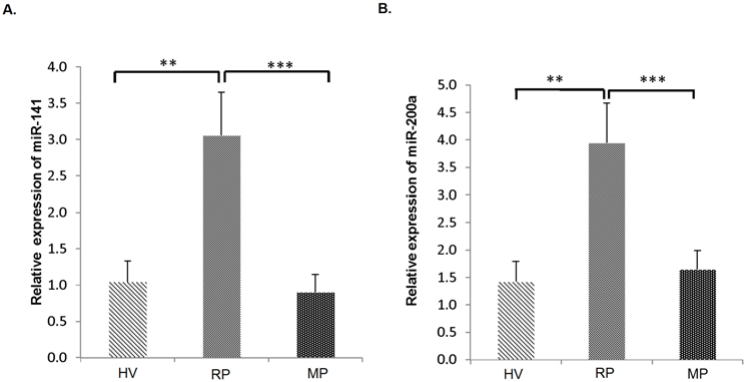
Up-regulation of miR-141 and miR-200a in relapsing phase of MS patients. RT-qPCR analysis of miR-141 expression level in CD4^**+**^ T cells of MS patients in relapsing phase (n = 20), remitting phase (n = 20) and healthy controls (n = 10) (*A*). RT-qPCR analysis of miR-200a expression level in CD4^**+**^ T cells of MS patients in the same groups (*B*). Results are normalized relative to expression level of reference gene, *RNU48* (*p < 0.05, **p < 0.01 and ***p < 0.005, non-parametric Mann-Whitney *t*-test) (RP: Relapsing patient, MP: Remitting patient, HV: Healthy volunteer).

Investigation of miR-141 expression level was carried out by RT-qPCR in three groups including relapsing and remitting phase of MS patients and healthy controls. Expression of miRNAs were normalized by small nucleolar RNA, *RNU48*, which was established to be the suitable reference gene beforehand [22]. miR-141 expression level was significantly higher in relapsing group compared to control and remitting groups (p value = 0.006 and p value = 0.001 respectively). Nevertheless, no meaningful difference was observed between control and remitting groups (p value = 0.837) (Fig 2A).

### Up-regulation of miR-200a in relapsing phase of MS and its similar expression pattern to miR-141

Analysis of miR-200a showed that miR-200a displays the same exact pattern as miR-141 expression. miR-200a expression level was significantly elevated in relapsing group versus control and remitting group (p value = 0.006 and p value = 0.003 respectively). There was also no significant difference between control and remitting group (p value = 0.741) (Fig 2B).

Primer specificity for miR-141, miR-200a and RNU48 was evaluated by poly acrylamide gel electrophoresis (S1 Fig). Additionally, sequencing results of cloned PCR products into pTZ57R/T (S2A–S2C Fig) confirmed the specificity of our primers.

### Expression analysis of master transcription factors involved in Th17/Treg differentiation along with *IL-17A* gene expression in CD4^+^ T cells

**Fig 3:**
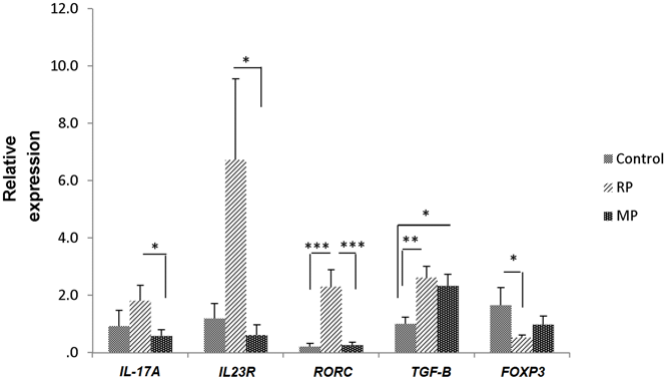
The expression level of master markers of Th17& Treg cells and *IL-17A* in CD4^+^ T cells of relapsing group (n = 20), remitting group (n = 20) and healthy controls (n = 10) in MS patients. Results are normalized relative to expression level of reference gene, *18srRNA* (*p < 0.05, **p < 0.01 and ***p < 0.005, non-parametric Mann-Whitney *t*-test) (RP: Relapsing patient, MP: Remitting patient, HV: Healthy volunteer).

To confirm the results of flow cytometry, we analyzed whether the expression level of master markers of Th17&Treg cells alter during the change of disease course from relapsing to remitting. The expression level of *IL-23R*, *RORC*, *TGF-β* and *FOXP3* were normalized to *18srRNA* as reference gene. Combined with this, we investigated the transcript level of *IL-17A* in relapsing group versus remitting and control groups. Expression level of *IL-17A* significantly increased in relapsing phase compared to remitting phase (p value = 0.023) but no significant differences were found between remitting with control and relapsing with control group (p value = 0.694 and p value = 0.329 respectively). *IL23R* expression displayed significant increase in relapsing versus remitting (p value = 0.042), however no significance was observed in remitting versus control and control versus relapsing (p value = 0.815 and p value = 0.058 respectively). Analyses of *RORC* expression demonstrated that there is a significant rise in relapsing group compared to both remitting and control group (p value = 0.0007 and p value = 0.002 respectively), but there was no significant difference between remitting and control groups (p value = 0.914). *TGF-β* expression level showed significant increase in relapsing versus control (p value = 0.008). *TGF-β* also up-regulated in remitting group versus control (p value = 0.029), however no meaningful variation was found between relapsing and remitting (p value = 0.573). Finally, the expression level of *FOXP3* significantly decreased in relapsing compared to control group (p value = 0.046) but there were no significant difference between relapsing with remitting and control with remitting (p value = 0.341 and p value = 0.187 respectively) (Fig 3).

### Molecular signaling pathway enrichment analysis of miR-141 and miR-200a targetome proposes possible inducing role of miR-141 and miR-200a in differentiation of Th17 cells

**Fig 4:**
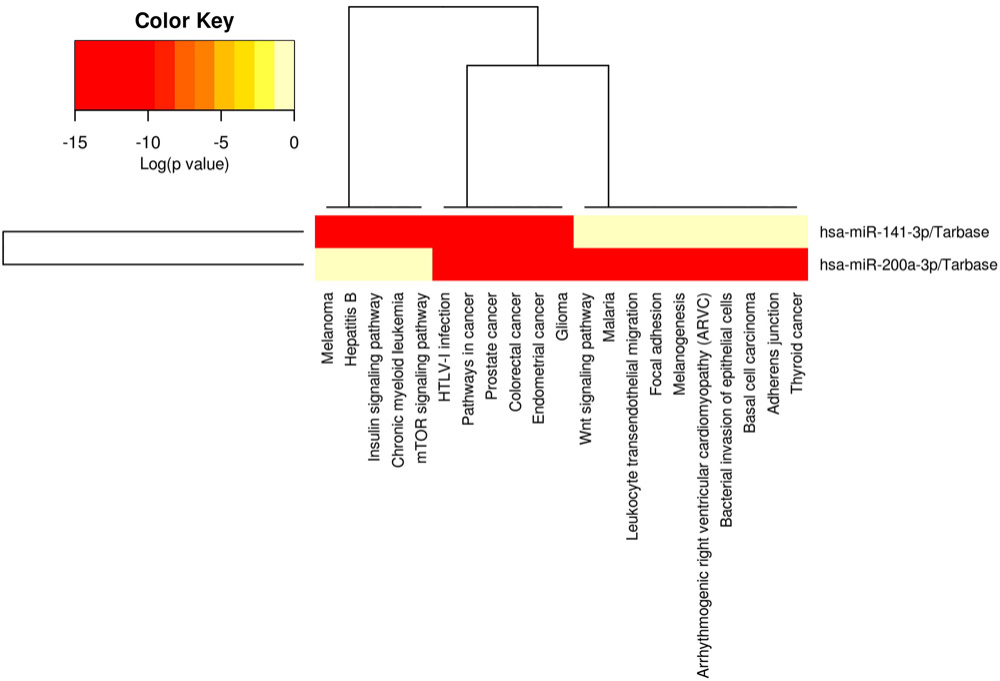
Heatmap view of pathways in which validated targets of miR-141 and miR-200a are involved. The heatmap was drawn in consequent of enrichment analysis on miR-141 and miR-200a targetome (for valid targets) in which a merged p-value is calculated for each pathway by applying Fisher’s meta-analysis manner. The resulting p-value represents the examined pathways that are significantly enriched with gene targets of miR-141 and miR-200a. Color gradient displays the importance of mentioned pathways. Heatmap was drawn based on validated targets of miR-141 and miR-200a, according to DIANA miRPath. Notably, mTOR signaling pathway was specified as one of the major pathways based on its involvement in differentiation of Th17 cells. Analysis shows that miR-141 affects mTOR pathway more effective than miR-200a does.

To recognize the possible role of miR-141 and miR-200a in Th17 differentiation, molecular signaling pathway enrichment analysis was conducted. Using miRWalk and miRTarBase databases, 2248 and 25 predicted and validated mRNAs for miR-141 and 2195 and 26 predicted and validated mRNAs for miR-200a were specified, respectively (S2A and S2B Table). All predicted targets in miRWalk were confirmed in at least five prediction databases. Additionally, all validated mRNA targets collected from miRTarBase are supported by experimental evidences including RT-qPCR, western blotting and reporter assay. Heatmap view of validated targets of miR-141 and miR-200a designed by DIANA miRPath v.2.0 showed that the only pathway involved in differentiation of Th17 cells which miR-141 and miR-200a might effectively influence is mTOR signaling pathway [23] (Fig 4). Additional examination displayed that 14 of validated targets of miR-141 and 17 of validated targets of miR-200a were expressed in lymph node and thymus by Unigene database (S2D and S2E Table).

Moreover, 14 mRNAs of all predicted targets for miR-141 and miR-200a that their roles in differentiation of T helper cells have been revealed in our studies were specified and taken into Unigene. The incoming results exhibited their expression in lymph node and thymus (S2D and S2E Table). Lastly, validated and predicted genes with defined expression in lymph node and thymus were chosen as miR-141 and miR-200a targetome for extra molecular enrichment analysis. Inputting official gene symbols of miR-141 and miR-200a targetome into the functional annotation tool of DAVID, a combined view of statistically meaningful pathways associated with above genes in BIOCARTA, KEGG and PANTHER was acknowledged (Table 1).

**Table 1:** Top statistically related signaling pathways to miR-141 and miR-200a targetome obtained from different databases by means of DAVID tool.

miR-141			miR-200a		
Category	Pathway	P value	Category	Pathway	P value
KEGG pathway	Pathways in cancer	1.3E-4	KEGG pathway	Pathways in cancer	3.2E-4
PANTHER pathway	JAK / STAT signaling pathway	3.4E-6	PANTHER pathway	JAK / STAT signaling pathway	4.5E-6
PANTHER pathway	TGF-β signaling pathway	6.8E-3	PANTHER pathway	TGF-β signaling pathway	8.7E-3
KEGG pathway	Chronic myeloid leukemia	1.0E-3	PANTHER pathway	Interleukin signaling pathway	2.6E-2
PANTHER pathway	EGF receptor signaling pathway	3.5E-2	PANTHER pathway	EGF receptor signaling pathway	4.1E-2
BIOCARTA	mTOR Signaling Pathway	1.9E-3	PANTHER pathway	PDGF signaling pathway	7.6E-2
KEGG pathway	Acute myeloid leukemia	1.1E-2	KEGG pathway	Endometrial cancer	1.1E-2
KEGG pathway	Insulin signaling pathway	5.2E-2	KEGG pathway	Chronic myeloid leukemia	2.3E-2
PANTHER pathway	Insulin/IGF pathway-protein kinase B signaling cascade	7.6E-2	KEGG pathway	Adherence junction	2.4E-2
BIOCARTA	Regulation of eIF4e and p70 S6 Kinase	2.2E-2	BIOCARTA	mTOR Signaling Pathway	3.1E-2
PANTHER pathway	Interleukin signaling pathway	9.5E-2	KEGG pathway	Colorectal cancer	2.8E-2
BIOCARTA	GATA3 participate in activating the Th2 cytokine genes expression	9.4E-2	PANTHER pathway	p53 pathway feedback loops 2	4.6E-2
KEGG pathway	Insulin signaling pathway	5.2E-2	KEGG pathway	Leukocyte transendothelial migration	5.2E-2
			PANTHER pathway	Ras Pathway	8.8E-2
			BIOCARTA	GATA3 participate in activating the Th2 cytokine genes	1.0E-1

**Fig 5:**
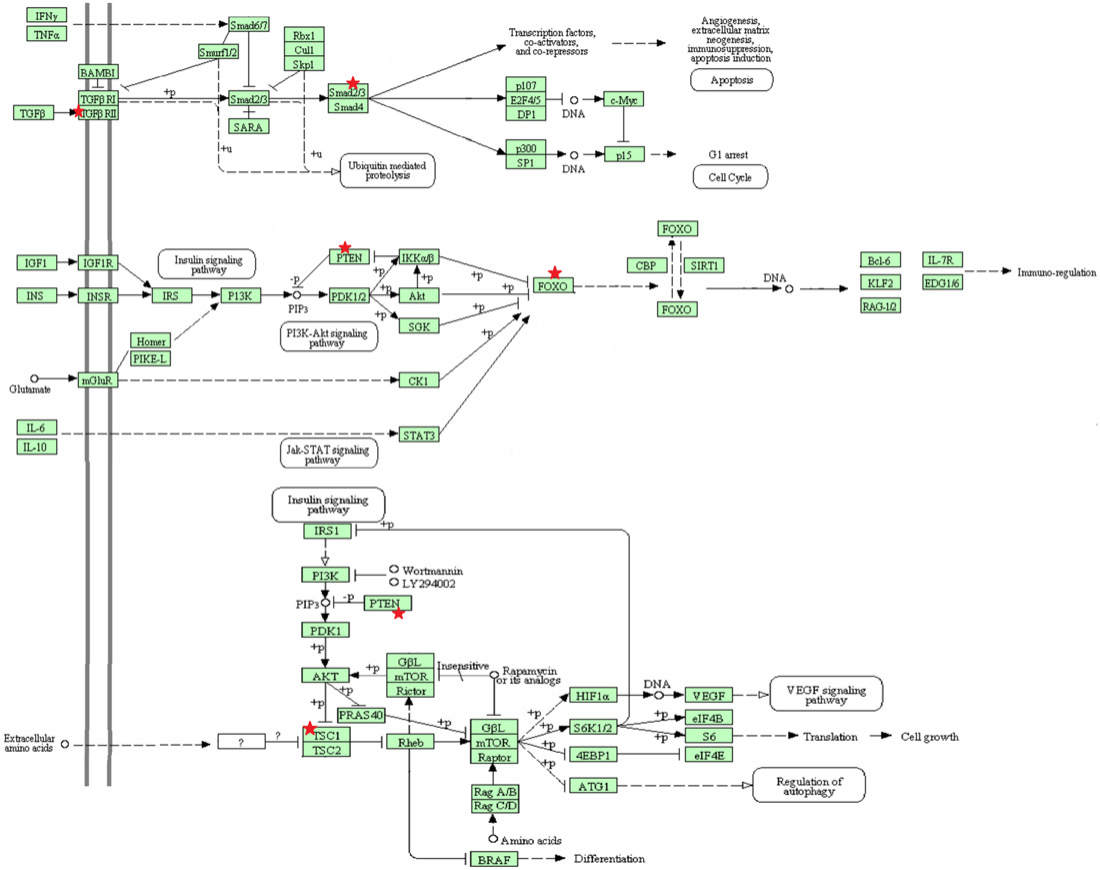
miR-141 and miR-200a targetome are both involved in several important pathways including TGF-β, mTOR and FOXO, which their partial diagram collected from KEGG pathway is demonstrated. Red stars mark common targets of miR-141 and miR-200a.

miR-141 and miR-200a targetome were commonly enriched in pathways in cancer, JAK/STAT signaling pathways and interestingly in mTOR and TGF-β pathways. Fig 5 demonstrates the combined intended schematic KEGG signaling pathways. Targets of miR-141 and miR-200a are indicated with red stars.

### Investigating the expression level of miR-141 and miR-200a’s common targets defined by molecular pathway enrichment analysis

**Fig 6:**
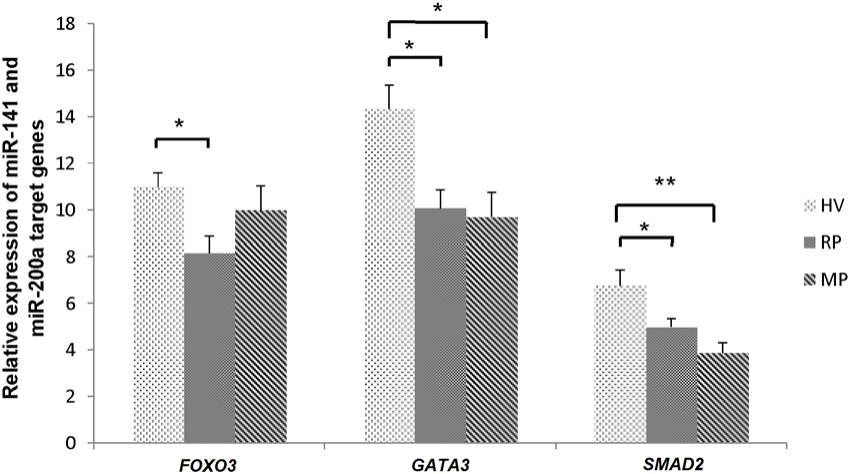
Target genes of miR-141 and miR-200a specified *in-silico* were tested for further analysis and the expression level of target genes of miR-141 and miR-200a in CD4^+^ T cells of relapsing group (n = 20), remitting group (n = 20) and healthy controls (n = 10) were assessed. Results are normalized relative to expression level of reference gene, *18srRNA* (*p < 0.05 and **p < 0.01, non-parametric Mann-Whitney *t*-test) (RP: Relapsing patient, MP: Remitting patient, HV: Healthy volunteer).

Next, to validate our *in-silico* experiments, several common targets of miR-141 and miR-200a such as: *FOXO3*, *SMAD2* and *GATA3* (not shown in the picture) were chosen for extra analyses of the effect of miR-141 and miR-200a on their targets. Interestingly results showed that expression level of *FOXO3* decreased significantly in relapsing compared to the control group (p value = 0.047). Expression level of *SMAD2* and *GATA3* displayed similar pattern in which their expression declined meaningfully in both relapsing and remitting groups compared to healthy group (p value = 0.028 and p value = 0.001 respectively for *SMAD2*, p value = 0.017 and p value = 0.010 respectively for *GATA3*) (Fig 6).

## Discussion

As mentioned before, Th17 and Treg cells are involved in pathogenesis of MS [4]. Recently, there are growing interest to unravel different miRNAs’ roles in various pathways involved in differentiation of these cells. However, the main challenge in this scenario is “how” and “which” miRNAs participate in differentiation of T helper cells. Several studies due to miRNAs in MS were conducted in PBMC, lymphocytes and/or other tissues, so far. These studies tried to find and nominate miRNAs with utmost importance as biomarkers and/or therapeutic solutions. Thus, introducing new miRNAs to the world of autoimmune diseases, especially MS and finding their possible role in the course of the disease is essential.

A number of studies explored the correlation between miR-141 and miR-200a with different autoimmune diseases such as SLE. Accordingly, miR-141 and miR-200a may contribute to other autoimmune diseases with the same immune-pathologic mechanism. Hence, we investigated the role of miR-141 and miR-200a in pathogenesis if MS.

In this study, we identified that miR-141 and miR-200a significantly elevated in relapsing compared to remitting group of MS and control group. In addition our flow cytometry results showed that percentage of Th17 cells is higher in relapsing group versus remitting and control groups while percentage of Treg cells increased in remitting group. Based on our results, we hypothesized that overexpression of miR-141 and miR-200a in relapsing compared to remitting phase of MS, regulate differentiation of Th17 cells while inhibiting differentiation to Treg cells.

Generally, Th17 cells have crucial roles in inducing autoimmunity in various autoimmune diseases. On the contrary, Treg cells inhibit autoimmunity. Studies on frequency of Th17 and Treg cells in peripheral blood in MS also illustrate that number of Th17 cells are found to be higher in relapsing phase of MS patients than either remitting or healthy controls while Treg cells are found at higher rate in healthy controls compared to MS phases [24,25]. As notified, no study evaluated the expression level of miR-141 and miR-200a in CD4^+^ T cells of MS patients so far, but a recent study conducted on B lymphocytes of MS patients showed that expression level of miR-200a in B lymphocytes of MS patients decreased compared to healthy controls [26] which seems rational as they inspected miRNA expression in B lymphocytes while the present study is focused on CD4^+^ T cells, especially Th17 and Treg cells. Further studies on miR-141 and miR-200a expression in other autoimmune-Th17 related diseases like idiopathic thrombocytopenic purpura (ITP) state that expression level of miR-141 was down regulated in PBMC of patients [27]. Since PBMC is a cocktail of different cells and expression of miRNAs are specific in each cell type, thus expression of miRNAs can be highly unpredictable and unreliable in PBMC. Another study by Olaru *et al*. showed up-regulation of miR-141 and miR-200a in IBD-dysplasia [28]. Also a study on serum and urinary free miRNAs of SLE patients displayed that both miR-141 and miR-200a down-regulated in serum and urine of patients [16]. Considering the fact that expression and presence of miRNAs in serum and urine are different from what is in the cell, we can vindicate our results. While writing this manuscript, we found a newly published study explaining the overexpression of miR-141 in brain tissues of EAE mice, suggesting new perceptions of the role of miRNAs as potential therapeutic agents. This is generally consistent with our findings [29].

In order to further confirm our results, we assessed the expression level of *IL-17A*, *IL23R*, *RORC*, *TGF-β* and *FOXP3* in different phases of MS. Our analyses showed up-regulation of *IL23R*, *RORC* and *IL-17A* in relapsing phase of MS patients.

IL-17A is one of the most important cytokines of Th17 cells and induce inflammatory reactions by stimulating the production of other cytokines and chemokine and its increase in serum and CSF of patients has been reported [10]. As, IL-23 is required for continuous differentiation of Th17 cells, *IL23R* can mainly be expressed in Th17 cells [30]. *RORC* gene transcribes to various isoforms including RORγt (RORC2) which is the master transcription factor of Th17 differentiation [31]. Therefore, our findings approve inclination of Th17 cells in relapsing phase of MS patients. Interestingly our data demonstrate that *TGF-β* level elevates in both relapsing and remitting phases of MS. This correlates with dual role of *TGF-β* in both differentiations of Th17 and Treg cells. Studies show that high concentrations of *TGF-β* leads to activation of *FOXP3*, thus differentiation to Treg cell while low concentrations of *TGF-β* leads to activation of *STAT3* and *RORγt*, thus differentiation to Th17 cell [32]. Giving this, up-regulation of *TGF-β* in both phases is correlated with Th17/Treg development in relapsing and remitting phases respectively. As mentioned earlier, *FOXP3* is the master transcription factor of Treg cells [8]. Expression level of *FOXP3* displays down regulation in relapsing phase compared to control group but no significant increase was found in remitting group, presumably due to either the fact that many genes are also regulated at the post-transcriptional level [33], hence measuring protein level seems to be more accurate, or limited number of patients and healthy controls in current study. Consequently, in agreement with our flow cytometry results, Th17 cells are increasing in relapsing while Treg cells are increasing in remitting phase of MS patients which confirm plausible contribution of miR-141 and miR-200a in differentiation of T helper cells.

Our computational analyses revealed that JAK/STAT, TGF-β and mTOR signaling pathways, combined with other pathways, are probably among the most important pathways affected by miR-141 and miR-200a. Trying to nominate multiple important genes which are direct targets of miR-141 and miR-200a and are also involved in differentiation of T helper cells, we selected *FOXO3*, *SMAD2* and *GATA3*.

*GATA3* is the master regulator of Th2 cells as studies showed that loss of *GATA3* in peripheral CD4^+^ T cells abolish differentiation to Th2 cells [8]. Moreover, it is now clear that *TGF-β* induce differentiation of both Th17 and Treg cells [32]. *TGF-β* acts through inducing a FOXP3^+^ RORγt^+^ double positive phenotype in naïve CD4^+^ T cells as well as inhibiting differentiation to Th1 and Th2 cells. It has been also stated that *SMAD2* and *SMAD3*, downstream genes in TGF-β pathway, are important in differentiation of Th17 cells [34]. Lastly, activation of mTORC2 leads to phosphorylation and suppression of *FOXO3* which is critical for appropriate development of Th17 cells. CD4^+^ T cells deficient in mTOR do not differentiate into either Th1, Th2 or Th17 cells [23,35]. Therefore inhibition of *FOXO3* together with other cited pathways leads the differentiation to Th17 cells. Our analyses also revealed that expression level of *SMAD2*, *GATA3* and *FOXO3* decline in relapsing phase of MS, concluding that their lessened expression level promote Th17 differentiation while inhibiting Treg differentiation. Collectively, our findings propose that miR-141 and miR-200a may possibly inhibit *SMAD2*, *GATA3* and *FOXO3* through which promote differentiation of Th17 cells and inhibition of Treg and/or other T helper subset developmental pathway.

In conclusion, in current study we indicated that miR-141 and miR-200a up-regulate in relapsing compared to remitting phase of MS patients and controls. In addition, we showed elevated percentage of Th17, simultaneous with reduced level of Treg cells in relapsing phase of MS. Thus, we proposed involvement of miR-141 and miR-200a in differentiation of T helper cells. Our *in-silico* analyses further approved that miR-141 and miR-200a may involve in pathways of Th17/Treg differentiation such as JAK/STAT, TGF-β and mTOR pathways. Finally, we also revealed declined expression level of *SMAD2*, *GATA3* and *FOXO3*, as direct targets of miR-141 and miR-200a in relapsing phase of MS. Thus, we suggest probable involvement of both miR-141 and miR-200a in differentiation of Th17 and Treg cells. Hence, these novel Th17-associated miRNAs could be investigated as potential therapeutic targets to inhibit MS progression in future. However, additional *in-vitro* and *in-vivo* experiments are also needed to further confirm this phenomenon.

## Supporting Information

S1 Fig Real time PCR products for miR-141, miR-200a and RNU48 were run on poly acrylamide gel and existence of only one band validates the specificity of our primers (M:50_bp_). (TIF)

S2 Fig One positive clone from PCR product of each miRNA was selected and extracted plasmids were sent for sequencing. Sequencing data analyses revealed that miR-141, miR-200a and RNU48 primers specifically amplified miR-141 (*A*), miR-200a (*B*) and RNU48 (*C*).
(TIF)

S1 Table Patient’s clinical appearances. (DOC)

S2 Table *In-silico* analyses on targets of miR-141 and miR-200a. Table A shows predicated and validated targets of miR-141, using miRWalk and miRTarBase databases respectively. Similar data are shown for miR-200a in Table B. Common targets of both miRs are shown separately in Table C. EST profiles of miR-141 and miR-200a which contain predicated and validated mRNA targets of miR-141 in thymus and lymph node are shown in Tables D and E respectively.
(XLSX)
