# Supplementary figures and images for: miR-141 and miR-200a, Revelation of New Possible Players in Modulation of Th17/Treg Differentiation and Pathogenesis of Multiple Sclerosis
Source: PLoS One. 2015 May 4;10(5):e0124555. doi: 10.1371/journal.pone.0124555 (PMC4418573; doi:10.1371/journal.pone.0124555)

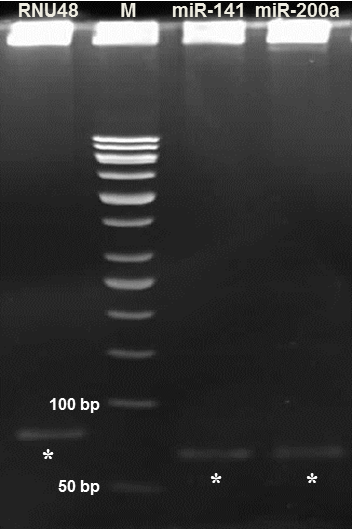

Supplement: S1 Fig — (TIF) [file pone.0124555.s001.tif]

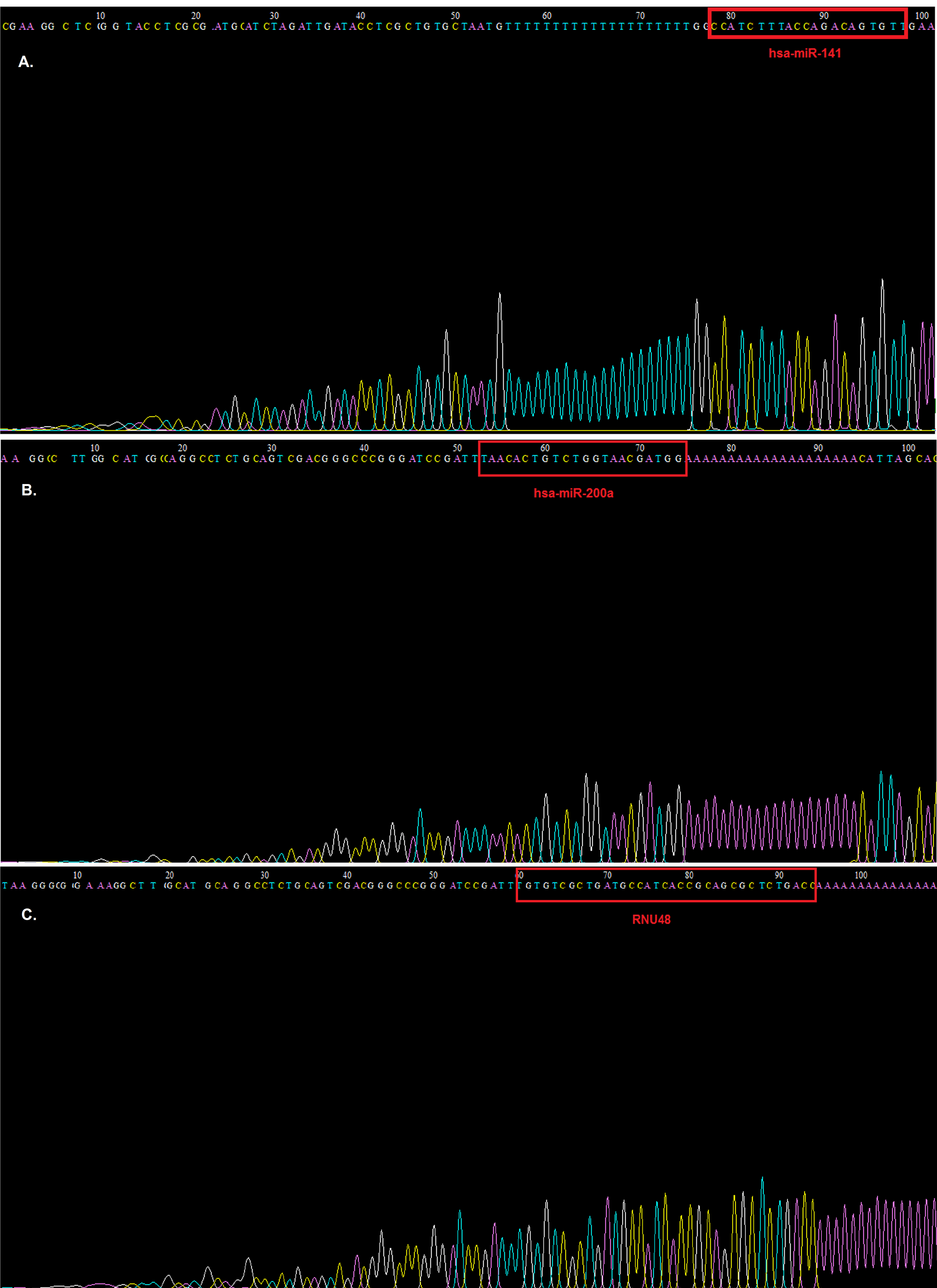

Supplement: S2 Fig — Sequencing data analyses revealed that miR-141, miR-200a and RNU48 primers specifically amplified miR-141 (A), miR-200a (B) and RNU48 (C). (TIF) [file pone.0124555.s002.tif]
