# Supplementary material for: miR-141 and miR-200a, Revelation of New Possible Players in Modulation of Th17/Treg Differentiation and Pathogenesis of Multiple Sclerosis
Source: PLoS One. 2015 May 4;10(5):e0124555. doi: 10.1371/journal.pone.0124555 (PMC4418573; doi:10.1371/journal.pone.0124555)
Supplement: S1 Table — (DOC) [file pone.0124555.s003.doc]

Supplementary Table 1. Patient’s clinical characteristics.

| **Patient number** | **Sex** | **Age (year)** | **Clinical phase** | **Disease duration (year)** | **MRI test results** |
| --- | --- | --- | --- | --- | --- |
| **1** | **F** | **28** | **Relapsing** | **NC** | **Ventricular plaques** |
| **2** | **F** | **31** | **Relapsing** | **NC** | **Several periventricular plaques** |
| **3** | **M** | **29** | **Relapsing** | **NC** | **3 to 4 periventricular plaques** |
| **4** | **F** | **27** | **Relapsing** | **NC** | **Several periventricular and corpus callosum plaques** |
| **5** | **M** | **22** | **Relapsing** | **NC** | **Several Periventricular plaques** |
| **6** | **F** | **34** | **Relapsing** | **NC** | **Ventricular plaques** |
| **7** | **F** | **25** | **Relapsing** | **NC** | **Cervical plaques** |
| **8** | **F** | **31** | **Relapsing** | **NC** | **Several little periventricular and cervical plaques** |
| **9** | **F** | **24** | **Relapsing** | **NC** | **Periventricular and spinal cord plaques** |
| **10** | **F** | **30** | **Relapsing** | **NC** | **Several Periventricular plaques** |
| **11** | **F** | **28** | **Relapsing** | **NC** | **Several Periventricular plaques** |
| **12** | **F** | **31** | **Relapsing** | **NC** | **Periventricular plaques** |
| **13** | **F** | **19** | **Relapsing** | **NC** | **Periventricular and spinal cord plaques** |
| **14** | **M** | **35** | **Relapsing** | **NC** | **Ventricular plaques** |
| **15** | **F** | **25** | **Relapsing** | **NC** | **Cervical plaques** |
| **16** | **F** | **28** | **Relapsing** | **NC** | **Periventricular plaques** |
| **17** | **M** | **22** | **Relapsing** | **NC** | **Cervical plaques** |
| **18** | **F** | **31** | **Relapsing** | **NC** | **Several periventricular and cervical plaques** |
| **19** | **F** | **26** | **Relapsing** | **NC** | **3 to 4 periventricular plaques** |
| **20** | **F** | **21** | **Relapsing** | **NC** | **Ventricular and spinal cord plaques** |
| **21** | **F** | **43** | **Remitting** | **1** | **Several periventricular and cervical plaques** |
| **22** | **F** | **23** | **Remitting** | **1.5** | **Periventricular plaques** |
| **23** | **M** | **24** | **Remitting** | **2** | **Several periventricular and cervical plaques** |
| **24** | **F** | **44** | **Remitting** | **8** | **Many periventricular plaques** |
| **25** | **F** | **32** | **Remitting** | **3** | **Periventricular plaques** |
| **26** | **F** | **41** | **Remitting** | **7** | **Several periventricular plaques** |
| **27** | **F** | **41** | **Remitting** | **10** | **Periventricular and spinal cords plaques** |
| **28** | **F** | **46** | **Remitting** | **12** | **7 to 6 periventricular and several cervical plaques** |
| **29** | **M** | **37** | **Remitting** | **2** | **Periventricular plaques** |
| **30** | **F** | **29** | **Remitting** | **1** | **Cervical and spinal cords plaques** |
| **31** | **F** | **51** | **Remitting** | **6** | **Many periventricular and several cervical plaques** |
| **32** | **F** | **36** | **Remitting** | **5** | **Periventricular plaques** |
| **33** | **F** | **28** | **Remitting** | **10** | **Periventricular and spinal cord plaques** |
| **34** | **M** | **31** | **Remitting** | **6** | **Several periventricular and cervical plaques** |
| **35** | **F** | **32** | **Remitting** | **2** | **5-6 periventricular plaques** |
| **36** | **M** | **39** | **Remitting** | **5** | **Periventricular and spinal cord plaques** |
| **37** | **F** | **31** | **Remitting** | **4** | **Periventricular plaques** |
| **38** | **F** | **33** | **Remitting** | **1** | **Periventricular plaques** |
| **39** | **M** | **37** | **Remitting** | **12** | **Many periventricular and several cervical plaques** |
| **40** | **M** | **32** | **Remitting** | **6** | **Cervical and spinal cords plaques** |

**(F = Female, M = male, NC = New Case)**
